# Long-term physical activity on prescription intervention for patients with insufficient physical activity level—a randomized controlled trial

**DOI:** 10.1186/s13063-020-04727-y

**Published:** 2020-09-15

**Authors:** Stefan Lundqvist, Mats Börjesson, Åsa Cider, Lars Hagberg, Camilla Bylin Ottehall, Johan Sjöström, Maria E. H. Larsson

**Affiliations:** 1 Department of Health and Rehabilitation, Unit of Physiotherapy, Institute of Neuroscience and Physiology, Sahlgrenska Academy, University of Gothenburg, Gothenburg, Sweden; 2 Centrum för fysisk aktivitet Göteborg, Gothenburg, Region Västra Götaland Sweden; 3 Center for Health and Performance (CHP), University of Gothenburg, Gothenburg, Sweden; 4 Institute of Medicine, Sahlgrenska Academy, University of Gothenburg, Gothenburg, Sweden; 5 Sahlgrenska University Hospital/Östra, Gothenburg, Region Västra Götaland Sweden; 6 University Health Care Research Center, Faculty of Medicine and Health, Örebro University, Örebro, Sweden; 7 Research and Development Primary Health Care, Gothenburg, Region Västra Götaland Sweden

**Keywords:** Primary health care, Physical activity, Metabolic syndrome, Quality of life, Health behaviour, Physical therapy

## Abstract

**Background:**

Physical activity (PA) can be used to prevent and treat diseases. In Sweden, licensed healthcare professionals use PA on prescription (PAP) to support patients to increase their PA level. The aim of this randomized controlled trial was to evaluate a 2-year intervention of two different strategies of PAP treatment for patients with insufficient PA level, after a previous 6-month period of ordinary PAP treatment in a primary health care setting.

**Methods:**

We included 190 patients, 27–77 years, physically inactive with metabolic risk factors where the patients were not responding to a previous 6-month PAP treatment with increased PA. The patients were randomized to either enhanced support from a physiotherapist (PT group) or continued ordinary PAP treatment at the health care centre (HCC group). The PAP treatment included an individualized dialogue; an individually dosed PA recommendation, including a written prescription; and a structured follow-up. In addition to PAP, the PT group received aerobic fitness tests and more frequent scheduled follow-ups. The patient PA level, metabolic health, and health-related quality of life (HRQOL) were measured at baseline and at 1- and 2-year follow-ups.

**Results:**

At the 2-year follow-up, 62.9% of the PT group and 50.8% of the HCC group had increased their PA level and 31.4% vs. 38.5% achieved ≥ 150 min of moderate-intensity PA/week (difference between groups n.s.). Over 2 years, both groups displayed increased high-density lipoproteins (HDL) (*p* = 0.004 vs. baseline), increased mental health status (MCS) (*p* = 0.036), and reduced body mass index (BMI) (*p* = 0.001), with no difference between groups.

**Conclusion:**

During long-term PAP interventions, the PA level, metabolic health, and HRQOL increased in patients at metabolic risk without significant differences between groups. The results indicate to be independent of any changes in pharmacological treatment. We demonstrated that the PAP treatment was feasible in ordinary primary care. Both the patients and the healthcare system benefitted from the improvement in metabolic risk factors. Future studies should elucidate effective long-term PAP-treatment strategies.

**Trial registration:**

ClinicalTrials.gov NCT03012516. Registered on 30 December 2016—retrospectively registered.

## Background

From 2007 to 2017, non-communicable diseases (NCDs) contributed to 3/4 of total deaths globally [1]. The most common cause of death was cardiovascular disease, and three metabolic risk factors, high systolic blood pressure (SBP), high fasting plasma glucose (FPG), and high body mass index (BMI), were considered leading contributors to the global burden of diseases [1, 2]. Physical inactivity was ranked the fourth leading risk factor for NCDs and global mortality [1, 3]. Robust evidence has supported the positive health effects of regular physical activity (PA) in humans, including the prevention and treatment of metabolic risk factors, cardiovascular disease, and diabetes type 2 [4]. Therefore, it is worrisome that only a minority of all adults are achieving the internationally recommended PA level of at least 150 min of moderate-intensity PA or 75 min of vigorous-intensity PA per week [3, 5].

Interventions that focus on increasing PA are highly important. A meta-analysis of predominately individual primary care, community, and home-based PA interventions in healthy adults found maintained improvement in PA levels for > 12 months compared to control group [6]. The evidence base for effectiveness, measured as standardized mean differences, reached levels of sufficiency and stability in 2011. In several countries, different variations of PA referral schemes have been tested in the healthcare system. Those studies produced varying results regarding, for example, PA levels, and further research is warranted [7, 8].

In Sweden, the physical activity on prescription (PAP) method include three core elements: patient-centred dialogues; individually-tailored PA recommendations, with a written prescription; and individualized, structured follow-ups. A recent systematic review presented high-level evidence, which showed that PAP can increase the PA level in patients being insufficiently active in the healthcare setting [9]. The individualized parts of PAP treatment may be crucial in influencing the patient’s capability and motivation to increase PA [10–12]. Swedish PAP studies have shown that, among subjects that increased their PA levels, compliance declined over time. At 6 and 12 months, 65% and 50% of patients, respectively, remained adherent to the recommended PA level [13, 14]. Rödjer et al. conducted a primary care PAP study and found that, compared to baseline, subjects reported significant increases in PA level at 6 and 12 months, followed by an on-going, but non-significant trend at 24 months [15].

All licensed Swedish healthcare professionals can use PAP treatment, but there is a need for more knowledge, clear, supportive management, and central/local supporting structures for the successful implementation of PAP treatment [11, 16]. These needs have also been highlighted internationally [17]. Healthcare professionals must provide intervention strategies that support both the initial change in behaviour and the maintenance of behavioural changes over time [18], because it is well known that establishing a new lifestyle habit takes time [19]. In Swedish health care, PA and PAP remain underutilized as treatment strategies [20], and further studies are needed to elucidate effective PAP treatment strategies, with longer follow-up periods, suitable for different patient groups [7, 21].

The present study conducted a 2-year, two-armed PAP trial on patients with insufficient PA level after a prior 6-month PAP treatment in a primary healthcare setting. The alternatives that we considered interesting to study was continued PAP treatment as before, in the health care centre (HCC), or an enhanced PAP treatment conducted by physiotherapist (PT).

The aim of this study was to explore possible differences between the PAP interventions concerning PA-level, metabolic risk factors and health-related quality of life and to evaluate the long-term effects of both methods.

## Methods

### Study design

This study was a 2-year randomized controlled trial (RCT) of PAP treatment with two arms: one PT group and one HCC group. The present study was part of an ongoing study that included 444 patients with a 5-year follow-up that was described previously [22, 23]. The study was approved by the Regional Ethical Review Board in Gothenburg, Sweden (Dnr 529-09).

### Study population

The study population included 190 patients, insufficiently physically active, according to the internationally recommended minimum PA level of 150 min/week, aged 27–77 years. With metabolic risk factors. Prior to study inclusion, the patients had undergone PAP treatment for 6 months at one of 15 designated health care centres in Gothenburg, Sweden. During the first 6 months of PAP treatment, 56% of patients increased their PA level to some extent, 22% decreased their PA level, and 22% remained unchanged. Importantly, all 190 patients, who were later included in the study, did not reach a PA level of 150 min/week. PA was assessed with two questions regarding moderate vs. vigorous PA intensity during the past week. All patients agreed to participate, both orally and in writing, and were included from 2010 to 2014. The patients were then randomized by an administrator (stratified randomization, based on block randomization). An automated computer-based programme stratified patients by age (≤ or > 55 years), sex (female or male), and BMI (< or ≥ 30 kg/m^2^). Patients were randomized to receive either enhanced PAP treatment, with support from a PT (PT group, *n* = 98), or continued ordinary PAP treatment, the same as the previous 6-month PAP treatment, with support from nurses at the HCC (HCC group, *n* = 92). The administrator communicated the results of the randomization to each patient and to the PT or HCC via telephone. The PT or HCC contacted the patient to begin the intervention.

### Intervention

Nurses trained in the health effects of PA, and the PAP method provided PAP treatment in the HCC group. The PAP treatment included an individualized dialogue about PA, an individually dosed PA recommendation, including a written prescription, and an individual-adjusted follow-up. The physiotherapists that provided PAP treatment in the PT group were also educated in PAP treatment. The intervention included the same first two parts of treatment described for the HCC group. The third part of treatment (the follow-up) differed and was arranged via a fixed follow-up schedule. Patients were followed up 6 times during the first year of the intervention (at 4 weeks, 10 weeks, 4 months, 6 months, 9 months, and 1 year) and three times during the second year (at 15 months, 18 months, and 2 years). The PT group also received an added aerobic physical fitness test (VO2max), performed on an ergometric bicycle (3 tests in the first year; 1 test in the second year). The results from the ergometer bicycle tests provided the basis for a continuing motivating dialogue about PA and an individually dosed PA recommendation. The agreed recommendations were written in the prescription regarding the appropriate frequency, duration, and intensity of PA. In summary, the RCT compared long-term, continual, standard PAP to enhanced PAP, with an added ergometer bicycle tests and more frequent follow-ups, according to a fixed schedule.

### Measurements

We measured the PA level, metabolic health, and health-related quality of life (HRQOL) in both groups, at baseline, at 1 year, and at 2 years. All measurements were performed by the nurses at the HCC. At baseline, we collected values for *age*, *sex*, *smoking*, *economic status*, *social situation*, and *education*. At the 1- and 2-year follow-ups, we measured *changes in medication during the prior 6 months* and the *frequency of PAP support from the healthcare provider at the HCC during the prior 6 months* where the patient’s contact frequency was categorized in 1–2, 3–5, 6–10, 11–20, and ≥ 21 contacts.

### PA level

PA was assessed with two questionnaires. The first questionnaire was a self-assessment of two PA questions. Patients received 1 point when they were physically active at a moderate-intensity level for 30 min per day, and 1.7 points when they were physically active at a more vigorous-intensity level for 20 min per day. A weekly score of ≥ 5 points indicated an adequate PA level, according to the American College of Sports Medicine (ACSM) and the American Heart Association (AHA) public health recommendations [24]. In this study, this questionnaire was referred to as the ACSM/AHA questionnaire. The second questionnaire was the International Physical Activity Questionnaire (IPAQ). It recorded the duration (min) and frequency (days) of three specific types of PA performed during the past 7 days: walking, moderate-intensity activities, and vigorous-intensity activities, which were scored separately. The results are presented as median metabolic equivalent (MET)-minutes per week (an energy expenditure estimate) with a total MET-minutes/week (TotalMET) summarized from the three types of PA, weighted as follows: walking (3.3 METs), moderate-intensity activity (4.0 METs), and vigorous-intensity activity (8.0 METs) (duration × frequency × MET intensity) [25, 26].

### Anthropometrics

For the BMI (kg/m^2^), body weight was measured with patient wearing light clothing and no shoes; it was estimated to the nearest 0.1 kg (electric scale Carl Lidén AFW D300, Jönköping, Sweden). Body height was measured in an upright position, without shoes; it was estimated to the nearest 0.5 cm (scale fixed to the wall, PEM 136, Hultafors, Sweden). The waist circumference (WC) was measured with the patient standing, after exhaling air from the lungs. A measuring-tape (Kirchner Wilhelm, Aspberg, Germany) was placed on the patient’s skin, between the lower rib and the iliac crest, and the WC was estimated to the nearest 0.5 cm.

### Blood pressure

Systolic and diastolic blood pressure (SBP, DBP) were measured (in mmHg) with the patient seated, after 5 min of rest [27]. The blood pressure sphygmomanometer (Omron HEM-907, Kyoto, Japan) was attached to the right upper arm at the level of the heart.

### Blood samples

Blood samples were analysed to determine the levels of fasting plasma glucose (FPG) after an overnight fast, triglycerides (TG), total cholesterol (Chol), high-density lipoprotein (HDL), and low-density lipoprotein (LDL), all expressed in mmol/litre. Values were analysed according to the European Accreditation system [28].

### Health-related quality of life

The Swedish version of the Short Form 36 (SF-36 Standard Swedish Version 1.0) was used to measure HRQOL [29]. The 36 questions generated eight health concepts, which were grouped to express the physical component summary (PCS) and the mental component summary (MCS). These scores were converted to a range of 0–100 points, where higher values represented a better HRQOL.

### Statistical analysis

Sample size was calculated based on a power of 87.5% to detect a difference of 20% between groups, in patients reaching ≥ 150 min of moderate-intensity PA/week, at a significance level of 0.05 [30]. We hypothesized that 40% in the PT group and 20% in the HCC group would reach a sufficient PA level. According to this analysis, 200 patients were needed: 100 patients in each group. We randomized 190 patients for the study, with 98 patients in the PT group and 92 patients in the HCC group.

Baseline characteristics are presented as the mean (±standard deviation [SD]), the median (25–75 percentiles), or the number (%). Baseline values were compared between the groups that completed 2 years in the study vs. the dropout group. Differences were evaluated with an independent sample *t* test or Mann-Whitney *U* test, according to data requirements.

All analyses were performed according to intention to treat (ITT). Missing data were, based on the authors’ knowledge of the data and research field, assumed be missing at random (MAR). Linear mixed-effects models were used to analyse longitudinal changes, from baseline to 1 or 2 years. The dependence between repeated measures for each individual was modelled by a random intercept, and the residuals were modelled with a diagonal covariance matrix, hence allowing for unequal variances at different time points. All parameters and marginal means for outcome variables were reported with point estimates and 95% confidence intervals (95% CI). Fixed effects independent variables were *time*, *group*, and the interaction term, *time × group*. Dependent variables were *TotalMET*, *BMI*, *WC*, *SBP*, *DBP*, *FPG*, *TG*, *Chol*, *HDL*, *LDL*, *PCS*, and *MCS*, respectively. To accomplish homogeneity of variances, we log-transformed the dependent variables: *TotalMET*, *FPG*, and *TG*. The potential covariates, *age*, *sex*, *smoking*, *economic status*, *social situation*, and *education*, at baseline were first added individually for each model. Interaction terms and potential covariates that showed *p* values > 0.05 were not included in the final regression model. Statistical significance was set at *p* value ≤ 0.05.

At the 2-year follow-up, we analysed increases in PA level using the ACSM/AHA questionnaire not included in the mixed effect-models analysis. We used the paired sample *t* test to evaluate within-group differences and the independent sample *t* test to evaluate between-group differences regarding increases in PA. These data were not shown in a table. The proportion of patients who achieved target PA level, according to public health recommendations, was presented in percent in each group. Additionally, at the 1- and 2-year follow-up, we analysed the follow-up question: *Have you changed your medication during the past 6 months?* with multiple choice options: *No*, *Yes increased*, or *Yes decreased.* We used the chi-square test for independence between groups and the McNemar-Bowker Test for within-group comparisons. Statistical significance was set at *p* ≤ 0.05.

## Results

### Study population

**Fig. 1:**
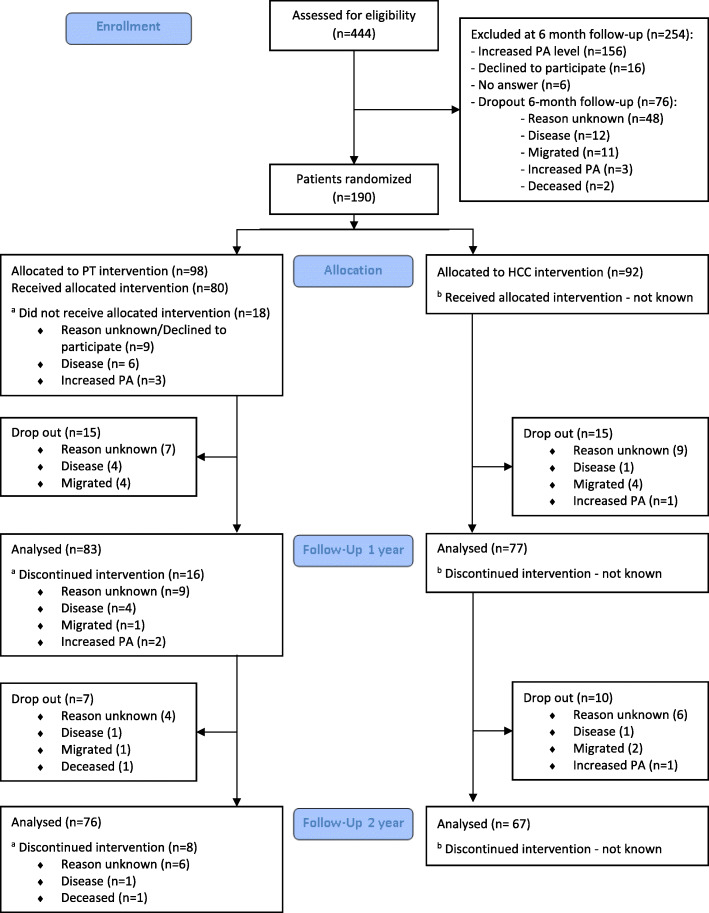
Flow of patients involved in the study. ^a^Majority of the patients in the PT-group not receiving allocated intervention or discontinuing intervention was attended to 1- and 2-year follow-up. ^b^The number of patients in the HCC group not receiving or discontinuing intervention is not known dependent on non-access to the patient’s medical record with the current information

Of the 98 patients randomized to the PT-group, 83 (85%) attended the 1-year follow-up and 64 (65%) had received and continued allocated intervention. At 2-year, 76 (78%) of the patients in the PT group attended the follow-up and 56 (57%) had continued allocated intervention (Fig. 1). In the HCC-group, out of 92 patients, 77 (84%) attended the 1-year follow-up and 67 (73%) attended the 2-year follow-up. Data on adherence to allocated intervention in the HCC group was uncertain due to the fact that we did not have access to the patient’s medical record with the current information (Fig. 1). However, in the questionnaire, a majority of patients in the HCC group answered that they received follow-up counselling with their PAP support caregiver 1–2 times during the 6 months prior to the 1-year (84%) and 2-year (92%) follow-ups.

### Baseline characteristics

The baseline characteristics are listed in Tables 1 and 2. The average PA level was low; the value corresponded to taking a brisk walk for 30 min, 2–3 times per week or less. The mean BMI was 32, and the mean WC were 113.6 cm for men and 105.2 cm for women (Table 2). A comparison of baseline characteristics between patients that attended the 2-year follow-up and the patients that dropped out of the study revealed no significant differences in PA level, metabolic risk factors, or HRQOL, except that the DBP was lower in the dropout group (difference = − 3.6 mmHg, *p* = 0.026; data not shown).

**Table 1:** Baseline characteristics of the patients in the physiotherapist and health care centre group

Variable	Total (*n* = 190)	PT group (*n* = 98)	HCC group (*n* = 92)
**Age**^a^, years	57 (10.8)	56.4 (10.2)	57.5 (11.3)
**Sex**^b^			
Female	94 (49.5)	48 (49.0)	46 (50.0)
Male	96 (50.5)	50 (51.0)	46 (50.0)
**Social situation**^b^			
Single	74 (40.4)	44 (46.3)	30 (34.1)
Married/cohabit	99 (54.1)	47 (49.5)	52 (59.1)
Other	10 (5.5)	4 (4.2)	6 (6.8)
**Economic status**^b^, perceived			
Good	110 (59.1)	56 (57.7)	54 (60.7)
Neither nor	46 (24.7)	26 (26.8)	20 (22.5)
Bad	30 (15.8)	15 (15.5)	15 (16.9)
**Education**^b^			
Elementary grade	35 (18.8)	16 (16.5)	19 (21.3)
Upper secondary school	62 (33.3)	37 (38.1)	25 (28.1)
University college	89 (47.8)	44 (45.4)	45 (50.6)
**Tobacco**^b^			
Smokers	21 (11.4)	9 (9.4)	12 (13.5)
Non-smokers	121 (65.4)	64 (66.7)	57 (64.0)
Ex-smokers	43 (23.2)	23 (24.0)	20 (22.5)
**Part of metabolic syndrome**^b^			
Overweight/obesity	167 (89.3)	88 (89.8)	79 (88.8)
Hyperglycaemia	68 (36.8)	35 (36.5)	33 (37.1)
Hypertension	150 (80.2)	75 (77.3)	75 (83.3)
Hyperlipidaemia	106 (57.0)	50 (52.1)	56 (62.2)
Other diagnosis			
Mental health, depression	24 (12.8)	16 (16.3)	8 (9.0)
Musculoskeletal disorders	25 (13.4)	15 (15.3)	10 (10.9)
Other	86 (46.0)	52 (53.1)	34 (38.2)
**Drug treatment**^b^			
Overweight/obesity	0 (0)	0 (0)	0 (0)
Hyperglycaemia	30 (16.1)	14 (14.4)	16 (18.0)
Hypertension	108 (57.8)	57 (58.8)	51 (56.7)
Hyperlipidaemia	42 (22.5)	23 (23.7)	19 (21.1)
Other drug treatment			
Mental health, depression	21 (11.2)	13 (13.3)	8 (9.0)
Musculoskeletal disorders	21 (11.2)	13 (13.3)	8 (8.7)
Other	67 (36.0)	39 (40.2)	28 (31.5)

Data are given as ^a^mean (standard deviation) or as ^b^number (percentage)

*PT* physiotherapist, *HCC* health care centre

**Table 2:** Baseline characteristics of the patients in the physiotherapist and health care centre group

Variable	Total (*n* = 190)	PT group (*n* = 98)	HCC group (*n* = 92)
Physical activity level			
ACSM/AHA questionnaire^a^, score	2.3 (1.5)	2.4 (1.4)	2.2 (1.6)
IPAQ^b^, total MET-minutes/week	792 (278–1672)	753 (198–1641)	822 (357–1742)
BMI^a^, kg/m^2^	32 (5.6)	32 (5.6)	32 (5.7)
Waist circumference^a^, cm	108.1 (14.2)	108.2 (14.3)	108.0 (14.3)
Blood pressure^a^, mm/Hg			
Systolic	133.6 (16.0)	132.6 (17.1)	134.7 (14.7)
Diastolic	82.4 (9.6)	82.2 (9.9)	82.5 (9.2)
Metabolic components^a^, mmol/l			
Fasting plasma glucose	6.1 (1.5)	6.1 (1.6)	6.1 (1.4)
Triglycerides	1.6 (0.8)	1.5 (0.8)	1.8 (0.8)
Cholesterol	5.4 (1.1)	5.4 (1.1)	5.3 (1.1)
HDL	1.4 (0.5)	1.4 (0.4)	1.4 (0.5)
LDL	3.4 (1.0)	3.5 (1.0)	3.4 (1.1)
HRQOL SF-36^a^, score			
Physical component summary	46.1 (10.8)	46.6 (11.3)	45.6 (10.2)
Mental component summary	44.3 (12.6)	42.7 (13.3)	46.1 (11.4)

Data are given as ^a^mean (standard deviation) or as ^b^median (25–75 percentiles)

*PT* physiotherapist, *HCC* health care centre, *ACSM* American College of Sports Medicine, *AHA* American Heart Association, *IPAQ* International Physical Activity Questionnaire, *MET* metabolic equivalent, *BMI* body mass index, *HDL* high-density lipoprotein, *LDL* low-density lipoprotein, *HRQOL SF-36* health-related quality of life 36-Item Short Form Health Survey

### Outcomes

Of the patients attending to the 2-year follow-up, 62.9% (*p* < 0.001) of the PT group and 50.8% (*p* < 0.001) of the HCC group had increased their PA level, according to the ACSM/AHA questionnaire. There were no significant differences in PA level between the groups at the 2-year follow-up (*p* = 0.785). At the 2-year follow-up, 31.4% of the PT group and 38.5% of the HCC group achieved the public health recommendation of at least ≥ 150 min of moderate-intensity PA/week. The dropout rate for completing the PA questionnaire was 29% in both groups.

**Fig. 2:**
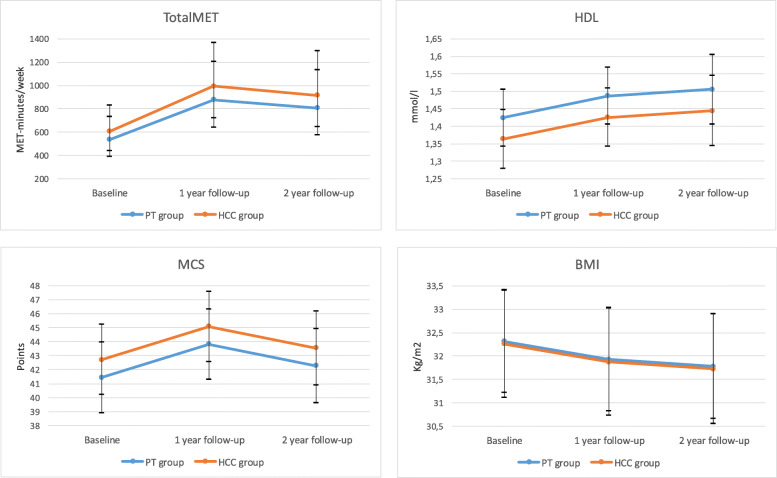
Physical activity level and health outcomes over time for the PT and HCC group^a^. ^a^Analysed with linear mixed effects models. TotalMET is presented with the estimated marginal geometric mean and 95% CI. HDL, MCS, and BMI are presented with the estimated marginal arithmetic mean and 95% CI. PT, physiotherapist; HCC, health care centre; MET, metabolic equivalent; CI, confidence interval; HDL, high-density lipoprotein; MCS, mental component summary; BMI, body mass index

Over the 2-year follow-up period, regression analyses showed no significant differences between the PT and HCC groups regarding the TotalMET scores, the metabolic risk factors, or the HRQOL (Table 3; Additional file 1). Compared to baseline levels, both the PT and HCC groups showed increases in the TotalMET scores (*p* = 0.002), HDL levels (*p* = 0.004), and MCS scores (*p* = 0.036), and a reduction in the BMI (*p* = 0.001) over the 2-year follow-up (Table 3; Fig. 2; Additional file 1).

**Table 3:** Summary of linear mixed effects model analysis

Outcome variable (*n*)	Independent variables ***p*** value*	
	Group	Time
	PT or HCC	Baseline, 1 year or 2 years
TotalMET^a^ (178)	0.532	**0.002**
BMI (188)	0.947	**0.003**
WC (189)	0.777	0.211
SBP (189)	0.703	0.211
DBP (189)	0.682	0.072
FPG^a^ (184)	0.930	0.997
TG^a^ (188)	0.072	0.167
Chol (188)	0.297	0.322
HDL (188)	0.287	**0.004**
LDL (188)	0.245	0.314
PCS (189)	0.400	0.780
MCS (184)	0.377	**0.036**

*MET* metabolic equivalent, *BMI* body mass index, *WC* waist circumference, *SBP* systolic blood pressure, *DBP* diastolic blood pressure, *FPG* fasting plasma glucose, *TG* triglycerides, *Chol* cholesterol, *HDL* high-density lipoprotein, *LDL* low-density lipoprotein, *PCS* physical component summary, *MCS* mental component summary

*Type III *F*-tests of fixed effects, testing whether the variable contributes significantly to the model

^a^Outcome variables were log transformed

Of potential covariates, older age had detrimental effects on the SBP and PCS and beneficial effects on the HDL and MCS. Women had more positive outcomes in WC, DBP, TG, and HDL, compared to males. Non-smokers had lower FPGs than smokers. Poor economic status negatively affected the MCS. (Additional file 1).

There were no between-group or within-group differences concerning changes in medication during the past 6 months, measured at the 1- and 2-year follow-up. A majority of patients had not changed their medication at the 1-year (77.3%) and 2-year (70.9%) follow-up (Additional file 2). The majority of the medication taken by the patients were medication for metabolic risk factors or non-communicable diseases, such as depression, anxiety, musculoskeletal disorders, asthma, and COPD.

## Discussion

The main findings of the present RCT were the improvements in PA level, metabolic risk factors, and HRQOL parameters during a long-term PAP treatment, with no differences between the two PAP methods. The study included patients remaining insufficiently physically active after a 6-month period of ordinary PAP treatment. After continuing PAP treatment for 2 years, a majority of patients in both the PT and HCC groups increased their PA level and improvements in TotalMET, BMI, HDL, and MCS over time were shown for both groups. Possibly the results would have been more pronounced if we had allowed the patients to choose the study arm, instead of randomly assigning them to the interventions as we acknowledge the importance of an individualized process for the patient [10–12]. The results should be considered in light of the fact that discontinuing PAP treatment at 6 months could risk adherence deterioration and a return to the baseline levels of PA and risk markers. More research is needed to investigate how PAP interventions might maintain PA involvement and behavioural changes over the long term [31].

It is important to evaluate different PAP treatment strategies. In this study, we found no differences in outcomes between continuing PAP treatment at the HCC and initiating PAP treatment supported by a PT. Both groups received largely the same treatment, in terms of individualized counselling and individually dosed PA recommendations, including a written prescription. However, the groups had different types of follow-up. The HCC group received individualized follow-ups, and the PT group received nine follow-up sessions that were scheduled at fixed times during the 2-year period. The absence of individualized follow-ups in the PT group might have affected the outcome, because individualization is thought to play a central role in the Swedish PAP model [9]. These flexible follow-ups are adapted to each patient’s need for support, and they have been considered essential among patients in Swedish healthcare [10]. The majority of the HCC group received individually customized follow-up counselling 1–2 times during the 6 months prior to the 1-year (84%) and 2-year (92%) follow-up. Thus, based on these results, a majority of patients received 4–8 counselling sessions during the 2-year follow-up period. The Swedish State’s Medical Assessment group (SBU) issued a report on methods for promoting PA where they stated that counselling could increase PA by 12–50%, and that increasing the counselling frequency over several months could increase the PA level even more [32]. In the present study, the groups showed little difference in counselling frequency during follow-up; thus, the difference in frequencies might be insufficient to affect a difference in outcomes between groups. Consequently, the added ergometer bicycle tests and more frequent, fixed follow-up sessions did not show any benefit compared to the standard PAP.

Apart from the significant beneficial changes in PA level, TotalMET scores, BMI, HDL, and MCS, compared to baseline, we found no significant detrimental changes in WC, SBP, DBP, FPG, TG, Chol, and LDL after the 2-year PAP intervention time (Additional file 1). This may be important since 42% of metabolically healthy abdominally obese individuals developed metabolic syndrome at 10 years [33]. Appleton et al. found that metabolically healthy obese subjects were more likely than healthy non-obese subjects to develop metabolic risk and diabetes type 2 during a 5–10-year period [34]. Achilike et al. concluded that metabolically healthy obese subjects were at high risk of developing multiple metabolic abnormalities [35]. Considering those findings, even the non-significant detrimental changes in metabolic risk factors in this study could be seen as a positive result. In this 2-year perspective of PAP treatment trial, there were both a treatment effect, where an increased PA level may improve the metabolic risk factors, and a preventive effect, where the improvement of risk factors is likely to reduce the incidence of future diseases. Consequently, improving the PA levels would also reduce mortality in a large patient group and preserve healthcare resources [36, 37].

A majority of patients had not changed their medication at the 1-year (77.3%) and 2-year (70.9%) follow-up, and there were no differences within or between the groups. A majority of the medication taken by the patients were medication for metabolic risk factors or non-communicable diseases, such as depression, anxiety, musculoskeletal disorders, asthma, and COPD, where increased PA has the possibility to positively affect all conditions. The results from the medication question indicates that the positive metabolic effects shown in this study was not primarily affected by an increase in medication.

The sample size calculation was based on the hypothesis that ≥ 150 min of moderate-intensity PA/week would be achieved by 40% in the PT group and 20% in the HCC group. However, we did not find any significant difference between the groups in patients achieving an adequate PA level. Perhaps, the fact that the patients continued the PAP intervention in both groups affected the PA level most, without additional effects from the relatively small extra support provided in the PT group.

### Strengths and limitations

The study has some limitations. The dropout rate between baseline and the 2-year follow-up was 22% in the PT group and 27% in the HCC group. It might have influenced the interpretation of results, due to a selection bias. However, the dropout rate was consistent with those reported in similar intervention studies [15, 38–40]. Of note, this study was a survey of daily clinical practice with no extra resources for the personnel to manage with the PAP-treatment routines. Of the patients in the PT group, 57% adhered to the allocated intervention during the 2-year study. However, the greatest loss of patients from the PT group was at baseline, where 18 patients (18%) declined to participate. We could not determine the proportion of patients in the HCC group that adhered to the allocated intervention, due to the fact that we did not have access to the patient’s medical record with the current information. Although uncertainties about patient dropout rates could influence the interpretation of results, under these circumstances, we expect that the results presented were probably not overestimated. Another potential limitation was that self-reported questionnaires for measuring PA might have resulted in overestimated activity levels, due to recall and response bias [41–43]. Sternfeld and Goldman-Rosas concluded that there is no single, perfect self-report measure but is nevertheless relevant to use in both research and practice settings, suited to an particular situation [44]. Both questionnaires used in this study measured “the previous week of PA”, something that is recommended by van Poppel et al. due to higher correlation to accelerometer data compared to measuring “the usual week of PA” [45]. Moreover, self-reported PA measures are frequently used, because they allow the collection of large amounts of data in a practical way, at low cost [46].

This study had several strengths. First, the PAP treatment was conducted by authorized personnel in an ordinary primary healthcare setting. Thus, the results had high external validity. Another strength was the use of linear mixed-effects models in the statistical analysis of longitudinal data and repeated measures. These models used all the available data, took the dependence structure in the data into account, and will, under the assumption of data MAR, yield unbiased parameter estimates. These features reduced the risk of type I and type II errors [47, 48]. Although the patients were randomized into two intervention groups, there was no control group of patients that received the usual care. This design complicated the interpretation of outcomes, given that the groups showed similar results. However, for both groups, the PAP intervention originated from a longitudinal observational study, where PAP treatment was part of the daily clinical practice; thus, we lacked the resources to organize a study design with three arms [22, 23]. Moreover, there were ethical arguments against randomizing patients from an ongoing PAP treatment to a control group without intervention. Establishing a new lifestyle habit takes time, and it would have been inappropriate to hinder motivated patients to continue a treatment, which in the long run, could positively affect their behaviour.

## Conclusions

This study demonstrated that during continued, long-term, 2-year intervention with two PAP treatment strategies, the PA level, metabolic health, and HRQOL increased in patients with insufficient PA level after a prior 6-month PAP treatment with no significant differences between groups. The results indicate to be independent of any changes in pharmacological treatment. The PAP treatment was feasible in an ordinary primary care setting, and the continuous support and duration of the intervention may be important factors for patients to increase and maintain PA. Improving metabolic risk factors benefits both the patients and the healthcare system. Further research is needed to evaluate the benefit of individualized long-term PAP treatment.

## Supplementary information

**Additional file 1.** Full model of linear mixed effects model analysis.

**Additional file 2.** Characteristics of the 1 and 2 year follow-up question: Have you changed your medication during the last 6 months?

## Data Availability

Data relevant to the study are included in the article and uploaded as supplementary information. The data that support the findings of this study are available from the corresponding author on reasonable request.

## Abbreviations

ACSM: American College of Sports Medicine
AHA: American Heart Association
BMI: Body mass index
Chol: Cholesterol
DBP: Diastolic blood pressure
FPG: Fasting plasma glucose
HCC: Health care centre
HDL: High-density lipoprotein
HRQOL: Health-related quality of life
ITT: Intention to treat
IPAQ: International Physical Activity Questionnaire
LDL: Low-density lipoprotein
LMEM: Linear mixed effects models
MCS: Mental component summary
MET: Metabolic equivalent
NCD: Non-communicable diseases
PA: Physical activity
PAP: Physical activity on prescription
PCS: Physical component summary
PT: Physiotherapist
RCT: Randomized controlled trial
SBP: Systolic blood pressure
SBU: Swedish State’s Medical Assessment
TG: Triglycerides
VO_2_ max: Maximal oxygen uptake
WC: Waist circumference

## References

[1] Global, regional, and national age-sex-specific mortality for 282 causes of death in 195 countries and territories, 1980–2017: a systematic analysis for the Global Burden of Disease Study 2017. Lancet. 2018;392(10159):1736–88. 10.1016/S0140-6736(18)32203-7. 10.1016/S0140-6736(18)32203-7 PMC6227606 30496103

[2] Global, regional, and national comparative risk assessment of 84 behavioural, environmental and occupational, and metabolic risks or clusters of risks for 195 countries and territories, 1990–2017: a systematic analysis for the Global Burden of Disease Study 2017. Lancet. 2018;392(10159):1923–94. 10.1016/S0140-6736(18)32225-6. 10.1016/S0140-6736(18)32225-6 PMC6227755 30496105

[3] World Health Organization (2010). WHO Guidelines Approved by the Guidelines Review Committee. Global Recommendations on Physical Activity for Health.

[4] King AC, Whitt-Glover MC, Marquez DX, Buman MP, Napolitano MA, Jakicic J (2019). Physical activity promotion: highlights from the 2018 physical activity guidelines advisory committee systematic review. Med Sci Sports Exerc.

[5] Lee IM, Shiroma EJ, Lobelo F, Puska P, Blair SN, Katzmarzyk PT (2012). Effect of physical inactivity on major non-communicable diseases worldwide: an analysis of burden of disease and life expectancy. Lancet.

[6] Love R, Adams J, van Sluijs EMF, Foster C, Humphreys D (2018). A cumulative meta-analysis of the effects of individual physical activity interventions targeting healthy adults. Obes Rev.

[7] Arsenijevic J, Groot W (2017). Physical activity on prescription schemes (PARS): do programme characteristics influence effectiveness? Results of a systematic review and meta-analyses. BMJ Open.

[8] Orrow G, Kinmonth AL, Sanderson S, Sutton S (2012). Effectiveness of physical activity promotion based in primary care: systematic review and meta-analysis of randomised controlled trials. BMJ (Clinical research ed).

[9] Onerup A, Arvidsson D, Blomqvist A, Daxberg EL, Jivegard L, Jonsdottir IH, et al. Physical activity on prescription in accordance with the Swedish model increases physical activity: a systematic review. Br J Sports Med. 2018. 10.1136/bjsports-2018-099598. 10.1136/bjsports-2018-099598 30413421

[10] Andersen P, Lendahls L, Holmberg S, Nilsen P (2019). Patients’ experiences of physical activity on prescription with access to counsellors in routine care: a qualitative study in Sweden. BMC Public Health.

[11] Bohman DM, Mattsson L, Borglin G (2015). Primary healthcare nurses’ experiences of physical activity referrals: an interview study. Prim Health Care Res Dev.

[12] Joelsson M, Bernhardsson S, Larsson ME (2017). Patients with chronic pain may need extra support when prescribed physical activity in primary care: a qualitative study. Scand J Prim Health Care.

[13] Kallings LV, Leijon ME, Kowalski J, Hellenius ML, Stahle A (2009). Self-reported adherence: a method for evaluating prescribed physical activity in primary health care patients. J Phys Act Health.

[14] Leijon ME, Bendtsen P, Ståhle A, Ekberg K, Festin K, Nilsen P (2010). Factors associated with patients self-reported adherence to prescribed physical activity in routine primary health care. BMC Fam Pract.

[15] Rodjer L, Ingibjörg HJ, Borjesson M (2016). Physical activity on prescription (PAP): self-reported physical activity and quality of life in a Swedish primary care population, 2-year follow-up. Scand J Prim Health Care.

[16] Gustavsson C, Nordqvist M, Broms K, Jerden L, Kallings LV, Wallin L (2018). What is required to facilitate implementation of Swedish physical activity on prescription? - interview study with primary healthcare staff and management. BMC Health Serv Res.

[17] Joy E, Blair SN, McBride P, Sallis R (2013). Physical activity counselling in sports medicine: a call to action. Br J Sports Med.

[18] Kwasnicka D, Dombrowski SU, White M, Sniehotta F (2016). Theoretical explanations for maintenance of behaviour change: a systematic review of behaviour theories. Health Psychol Rev.

[19] Marcus BH, Dubbert PM, Forsyth LH, McKenzie TL, Stone EJ, Dunn AL (2000). Physical activity behavior change: issues in adoption and maintenance. Health Psychol.

[20] Borjesson M (2012). Health care services can boost physical activity on prescription--more people need prescriptions. Lakartidningen.

[21] Sallis R, Franklin B, Joy L, Ross R, Sabgir D, Stone J (2015). Strategies for promoting physical activity in clinical practice. Prog Cardiovasc Dis.

[22] Lundqvist S, Borjesson M, Larsson ME, Hagberg L, Cider A (2017). Physical Activity on Prescription (PAP), in patients with metabolic risk factors. A 6-month follow-up study in primary health care. PLoS One.

[23] Lundqvist S, Börjesson M, Larsson MEH, Cider Å, Hagberg L (2019). Which patients benefit from physical activity on prescription (PAP)? A prospective observational analysis of factors that predict increased physical activity. BMC Public Health.

[24] Haskell WL, Lee IM, Pate RR, Powell KE, Blair SN, Franklin BA (2007). Physical activity and public health: updated recommendation for adults from the American College of Sports Medicine and the American Heart Association. Med Sci Sports Exerc.

[25] Ekelund U, Sepp H, Brage S, Becker W, Jakes R, Hennings M (2006). Criterion-related validity of the last 7-day, short form of the International Physical Activity Questionnaire in Swedish adults. Public Health Nutr.

[26] Craig CL, Marshall AL, Sjostrom M, Bauman AE, Booth ML, Ainsworth BE (2003). International physical activity questionnaire: 12-country reliability and validity. Med Sci Sports Exerc.

[27] O'Brien E, Asmar R, Beilin L, Imai Y, Mallion JM, Mancia G (2003). European Society of Hypertension recommendations for conventional, ambulatory and home blood pressure measurement. J Hypertens.

[28] European co-operation for Accreditation EA. [homepage on the Internet]. Available from: http://www.european-accreditation.org/ [updated 2016 May 17].

[29] Sullivan M, Karlsson J, Ware JE (1995). The Swedish SF-36 Health Survey--I. Evaluation of data quality, scaling assumptions, reliability and construct validity across general populations in Sweden. Soc Sci Med.

[30] McCrum-Gardner E (2010). Sample size and power calculations made simple. Int J Ther Rehabil.

[31] Biddle S, Mutrie N, Gorely T (2015). Psychology of physical activity: determinants, well-being and interventions.

[32] Hellenius M, Emtner M, Hagberg L, Johansson M, Kallings L, Lindahl B (2006). Metoder för att främja fysisk aktivitet-en systematisk litteraturöversikt. Statens beredning för medicinsk utvärdering (SBU).: Rapport nr 181.

[33] Eshtiaghi R, Keihani S, Hosseinpanah F, Barzin M, Azizi F (2015). Natural course of metabolically healthy abdominal obese adults after 10 years of follow-up: the Tehran Lipid and Glucose Study. Int J Obes (2005).

[34] Appleton SL, Seaborn CJ, Visvanathan R, Hill CL, Gill TK, Taylor AW (2013). Diabetes and cardiovascular disease outcomes in the metabolically healthy obese phenotype: a cohort study. Diabetes Care.

[35] Achilike I, Hazuda HP, Fowler SP, Aung K, Lorenzo C (2015). Predicting the development of the metabolically healthy obese phenotype. Int J Obes (2005).

[36] Guh DP, Zhang W, Bansback N, Amarsi Z, Birmingham CL, Anis AH (2009). The incidence of co-morbidities related to obesity and overweight: a systematic review and meta-analysis. BMC Public Health.

[37] World Health Organization. Global health risks: mortality and burden of disease attributable to selected major risks: World Health Organization; 2009. Available from: https://apps.who.int/iris/handle/10665/44203.

[38] Kallings LV, Leijon M, Hellenius ML, Stahle A (2008). Physical activity on prescription in primary health care: a follow-up of physical activity level and quality of life. Scand J Med Sci Sports.

[39] Morén C, Welmer A-K, Hagströmer M, Karlsson E, Sommerfeld DK (2016). The effects of “physical activity on prescription” in persons with transient ischemic attack: a randomized controlled study. J Neurol Phys Ther.

[40] Anderson RT, King A, Stewart AL, Camacho F, Rejeski WJ (2005). Physical activity counseling in primary care and patient well-being: do patients benefit?. Ann Behav Med.

[41] Hagstromer M, Ainsworth BE, Oja P, Sjostrom M (2010). Comparison of a subjective and an objective measure of physical activity in a population sample. J Phys Act Health.

[42] Lee PH, Macfarlane DJ, Lam TH, Stewart SM (2011). Validity of the International Physical Activity Questionnaire Short Form (IPAQ-SF): a systematic review. Int J Behav Nutr Phys Act.

[43] Shephard RJ (2003). Limits to the measurement of habitual physical activity by questionnaires. Br J Sports Med.

[44] Sternfeld B, Goldman-Rosas L (2012). A systematic approach to selecting an appropriate measure of self-reported physical activity or sedentary behavior. J Phys Act Health.

[45] van Poppel MN, Chinapaw MJ, Mokkink LB, van Mechelen W, Terwee CB (2010). Physical activity questionnaires for adults: a systematic review of measurement properties. Sports Med (Auckland, NZ).

[46] Sallis JF, Saelens BE (2000). Assessment of physical activity by self-report: status, limitations, and future directions. Res Q Exerc Sport.

[47] Brauer M, Curtin JJ (2018). Linear mixed-effects models and the analysis of nonindependent data: a unified framework to analyze categorical and continuous independent variables that vary within-subjects and/or within-items. Psychol Methods.

[48] Maurissen JP, Vidmar TJ (2017). Repeated-measure analyses: which one? A survey of statistical models and recommendations for reporting. Neurotoxicol Teratol.

