# Supplementary material for: Long-term physical activity on prescription intervention for patients with insufficient physical activity level—a randomized controlled trial
Source: Trials. 2020 Sep 15;21:793. doi: 10.1186/s13063-020-04727-y (PMC7493144; doi:10.1186/s13063-020-04727-y)
Supplement: Supplementary file 1 — Additional file 1. Full model of linear mixed effects model analysis. [file 13063_2020_4727_MOESM1_ESM.doc]

**Additional file 1**

| **Full model of linear mixed effects model analysis.** | | | | |
| --- | --- | --- | --- | --- |
| **Variable** | **Covariates** | **Parameter estimate** | **95 % CI** | ***p*-value*** |
| **TotalMET^a^** | Intercept | 6.40 | 6.09;6.72 |  |
|  | Time |  |  | **0.002** |
|  | 1 year | 0.50 | 0.22;0.78 |  |
|  | 2 year | 0.42 | 0.10;0.72 |  |
|  | Baseline | 0^b^ |  |  |
|  | Group |  |  | 0.532 |
|  | PT group | -0.12 | -0.52;0.27 |  |
|  | HCC group | 0^b^ |  |  |
| **BMI** | Intercept | 32.32 | 31.10;33.54 |  |
|  | Time |  |  | **0.003** |
|  | 1 year | -0.39 | -0.66;-0.12 |  |
|  | 2 year | -0.54 | -0.89;-0.19 |  |
|  | Baseline | 0^b^ |  |  |
|  | Group |  |  | 0.947 |
|  | PT group | 0,54 | -1.53;1.64 |  |
|  | HCC group | 0^b^ |  |  |
|  |  |  |  |  |
|  |  |  |  |  |
|  |  |  |  |  |
|  |  |  |  |  |
| **WC** | Intercept | 103.79 | 100.19;107.40 |  |
|  | Time |  |  | 0.211 |
|  | 1 year | -0.61 | -1.50;0.28 |  |
|  | 2 year | -0.80 | -1.76;0.16 |  |
|  | Baseline | 0^b^ |  |  |
|  | Group |  |  | 0.777 |
|  | PT group | -0.53 | -4.24;3.17 |  |
|  | HCC group | 0^b^ |  |  |
|  | Sex |  |  | **<0.001** |
|  | Male | 9.64 | 5.93;13.34 |  |
|  | Female | 0^b^ |  |  |
|  |  |  |  |  |
|  |  |  |  |  |
|  |  |  |  |  |
|  |  |  |  |  |
| **SBP** | Intercept |  |  |  |
|  | Time |  |  | 0.211 |
|  | 1 year | -2.36 | -4.63;-0.08 |  |
|  | 2 year | -1.15 | -3.87;1.57 |  |
|  | Baseline | 0^b^ |  |  |
|  | Group |  |  | 0.703 |
|  | PT group | -0.73 | -4.50;3.04 |  |
|  | HCC group | 0^b^ |  |  |
|  | Age | 0.34 | 0.16;0.51 | **<0.001** |
| **DBP** | Intercept | 80.86 | 78.76;82.95 |  |
|  | Time |  |  | 0.072 |
|  | 1 year | -1.32 | -2.83;0.19 |  |
|  | 2 year | -1.78 | -3.44;-0.13 |  |
|  | Baseline | 0^b^ |  |  |
|  | Group |  |  | 0.682 |
|  | PT group | -0.46 | -2.70;1.77 |  |
|  | HCC group | 0^b^ |  |  |
|  | Sex |  |  | **0.003** |
|  | Male | 3.42 | 1.18;5.65 |  |
|  | Female | 0^b^ |  |  |
| **FPG^a^** | Intercept | 1.75 | 1.70;1.80 |  |
|  | Time |  |  | 0.997 |
|  | 1 year | <0.001 | -0.03;0.03 |  |
|  | 2 year | -0.001 | -0.04;0.04 |  |
|  | Baseline | 0^b^ |  |  |
|  | Group |  |  | 0.930 |
|  | PT group | 0.003 | -0.06;0.60 |  |
|  | HCC group | 0^b^ |  |  |
|  | Smoking |  |  | **0.008** |
|  | Yes | 0.15 | -0.05;0.24 |  |
|  | Previous | 0.06 | -0.01:0.13 |  |
|  | No | 0^b^ |  |  |
| **TG^a^** | Intercept | 0.51 | 0.17;0.86 |  |
|  | Time |  |  | 0.167 |
|  | 1 year | -0.05 | -0.11;0.01 |  |
|  | 2 year | -0.04 | -0.10;0.02 |  |
|  | Baseline | 0^b^ |  |  |
|  | Group |  |  | 0.072 |
|  | PT group | -0.11 | -0.22;0.01 |  |
|  | HCC group | 0^b^ |  |  |
|  | Sex |  |  | **0.001** |
|  | Male | 0.21 | 0.09;0.33 |  |
|  | Female | 0^b^ |  |  |
| **Chol** | Intercept | 5.27 | 5.05;5.50 |  |
|  | Time |  |  | 0.322 |
|  | 1 year | -0.10 | -0.25;0.05 |  |
|  | 2 year | -0.11 | -0.27;0.05 |  |
|  | Baseline | 0^b^ |  |  |
|  | Group |  |  | 0.297 |
|  | PT group | 0.16 | -0.14;0.45 |  |
|  | HCC group | 0^b^ |  |  |
| **HDL** | Intercept | 1.05 | 0.72;1.39 |  |
|  | Time |  |  | **0.004** |
|  | 1 year | 0.06 | 0.02;0.10 |  |
|  | 2 year | 0.08 | 0.01;0.15 |  |
|  | Baseline | 0^b^ |  |  |
|  | Group |  |  | 0.287 |
|  | PT group | 0.06 | -0.05;0.18 |  |
|  | HCC group | 0^b^ |  |  |
|  | Sex |  |  | **<0.001** |
|  | Male | -0.30 | -0.42;-0.19 |  |
|  | Female | 0^b^ |  |  |
|  | Age | 0.01 | 0.002:0.01 | **0.004** |
| **LDL** | Intercept | 3.37 | 3.16;3.58 |  |
|  | Time |  |  | 0.314 |
|  | 1 year | -0.04 | -0.16;0.08 |  |
|  | 2 year | -0.11 | -0.25;0.04 |  |
|  | Baseline | 0^b^ |  |  |
|  | Group |  |  | 0.245 |
|  | PT group | 0.16 | -0.11;0.43 |  |
|  | HCC group | 0^b^ |  |  |
| **PCS** | Intercept | 59.08 | 50.85;67.30 |  |
|  | Time |  |  | 0.780 |
|  | 1 year | 1.76 | -0.38;3.91 |  |
|  | 2 year | -0.08 | -2.51;2.34 |  |
|  | Baseline | 0^b^ |  |  |
|  | Group |  |  | 0.400 |
|  | PT group | 0.34 | -2.75;3.43 |  |
|  | HCC group | 0^b^ |  |  |
|  | Age | -0.25 | -0.38;-0.12 | **<0.001** |
|  |  |  |  |  |
|  |  |  |  |  |
|  |  |  |  |  |
|  |  |  |  |  |
| **MCS** | Intercept | 27.23 | 18.85;35.62 |  |
|  | Time |  |  | **0.036** |
|  | 1 year | 2.38 | 0.54;4.23 |  |
|  | 2 year | 0.84 | -1.17;2.85 |  |
|  | Baseline | 0^b^ |  |  |
|  | Group |  |  | 0.377 |
|  | PT group | -1.28 | -4.13;1.57 |  |
|  | HCC group | 0^b^ |  |  |
|  | Economy |  |  | **0.001** |
|  | Good | 2.51 | -0.68;5.71 |  |
|  | Bad | -6.54 | -11.72;-1.37 |  |
|  | Neither or | 0^b^ |  |  |
|  | Age | 0.30 | 0.16;0.43 | **<0.001** |
| * type III F-tests of fixed effects, testing whether the variable as a whole contributes significantly to the model.  ^a^ Outcome variables were log transformed.  ^b^ This parameters were set to zero as they are redundant.  CI, confidence interval; MET, metabolic equivalent; PT, physiotherapist; HCC, health care centre; BMI, body mass index; WC, waist circumference; SBP, systolic blood pressure; DBP, diastolic blood pressure; FPG, fasting plasma glucose; TG, triglycerides; Chol, cholesterol; HDL, high density lipoprotein; LDL, low density lipoprotein; PCS, physical component summary; MCS, mental component summary. | | | | |
