# Supplementary material for: Long-term physical activity on prescription intervention for patients with insufficient physical activity level—a randomized controlled trial
Source: Trials. 2020 Sep 15;21:793. doi: 10.1186/s13063-020-04727-y (PMC7493144; doi:10.1186/s13063-020-04727-y)
Supplement: Supplementary file 2 — Additional file 2. Characteristics of the 1 and 2 year follow-up question: Have you changed your medication during the last 6 months? [file 13063_2020_4727_MOESM2_ESM.doc]

**Additional file 2**

| **Characteristics of the 1 and 2 year follow-up question: Have you changed your medication during the last 6 months?** | | | | |
| --- | --- | --- | --- | --- |
| **Variable** **(n)** | **Total** | **PT group** | **HCC group** | ***p* value** |
|  |  |  |  |  |
| 1 year follow-up (132) |  |  |  | 0.524 |
| No | 102 (77.3) | 52 (74.3) | 50 (80.6) |  |
| Increased | 15 (11.4) | 8 (11.4) | 7(11.3) |  |
| Decreased | 15 (11.4) | 10 (14.3) | 5 (8.1) |  |
|  |  |  |  |  |
| 2 year follow-up (117) |  |  |  | 0.236 |
| No | 83 (70.9) | 39 (65.0) | 44 (77.2) |  |
| Increased | 23 (19.7) | 13 (21.7) | 10 (17.5) |  |
| Decreased | 11 (9.4) | 8 (13.3) | 3 (5.3) |  |
|  |  |  |  |  |
| Data are given as number (percentage).  Difference between PT and HCC group. *P*-value was determined by a Chi-square test for independence. Statistical significance was set at *p*≤ 0.05.  PT, physiotherapist; HCC, health care centre. | | | | |
